# Characterization of the *REVEILLE* family in Rosaceae and role of *PbLHY* in flowering time regulation

**DOI:** 10.1186/s12864-023-09144-4

**Published:** 2023-01-28

**Authors:** Zhe Liu, Xiaoxuan Zhu, Weijuan Liu, Kaijie Qi, Zhihua Xie, Shaoling Zhang, Juyou Wu, Peng Wang

**Affiliations:** 1 Department of Pharmacy, Changzhi Medical College, Changzhi, 046000 China; 2 Sanya Institute of Nanjing Agricultural University, State Key Laboratory of Crop Genetics and Germplasm Enhancement, College of Horticulture, Nanjing Agricultural University, Nanjing, 210095 China; 3 Shanxi Province Key Laboratory of Functional Food with Homologous of Medicine and Food, Changzhi, China; 4 Jiangsu Key Laboratory for Horticultural Crop Genetic Improvement, Nanjing, China

**Keywords:** *Pyrus bretschneideri*, Flowering time, Circadian clock, RVE, LHY

## Abstract

**Background:**

The circadian clock integrates endogenous and exogenous signals and regulates various physiological processes in plants. REVEILLE (RVE) proteins play critical roles in circadian clock system, especially CCA1 (CIRCADIAN CLOCK ASSOCIATED 1) and LHY (LATE ELONGATED HYPOCOTYL), which also participate in flowering regulation. However, little is known about the evolution and function of the RVE family in Rosaceae species, especially in *Pyrus bretschneideri*.

**Results:**

In this study, we performed a genome-wide analysis and identified 51 *RVE* genes in seven Rosaceae species. The RVE family members were classified into two groups based on phylogenetic analysis. Dispersed duplication events and purifying selection were the main drivers of evolution in the *RVE* family. Moreover, the expression patterns of ten *PbRVE* genes were diverse in *P. bretschneideri* tissues. All *PbRVE* genes showed diurnal rhythms under light/dark cycles in *P. bretschneideri* leaves. Four *PbRVE* genes also displayed robust rhythms under constant light conditions. *PbLHY*, the gene with the highest homology to *AtCCA1* and *AtLHY* in *P. bretschneideri*, is localized in the nucleus. Ectopic overexpression of *PbLHY* in *Arabidopsis* delayed flowering time and repressed the expression of flowering time-related genes.

**Conclusion:**

These results contribute to improving the understanding and functional research of *RVE* genes in *P. bretschneideri*.

**Supplementary Information:**

The online version contains supplementary material available at 10.1186/s12864-023-09144-4.

## Background

The circadian clock regulates most of the metabolic and developmental processes of plants and synchronizes plants with external environmental conditions, which greatly enhances the adaptability and competitiveness of plants [1, 2]. The circadian clock system is composed of three parts: the input pathway, core oscillator, and output pathways [3]. Specifically, photoreceptors transmit environmental signals such as light and temperature to the core oscillator [4, 5]. Then, the core oscillator integrates information to regulate various physiological processes, such as flowering time, hormone signaling, biotic and abiotic stress [6]. The core oscillator mainly consists of three interlocked feedback loops, including a core loop and two closely related loops (morning loop and evening loop) [2]. CCA1 (CIRCADIAN CLOCK ASSOCIATED 1) and LHY (LATE ELONGATED HYPOCOTYL) are important components of the core loop [7]. In recent years, *CCA1* and *LHY*, as hub genes, have attracted increasing attention.

In *Arabidopsis*, *CCA1* is the first circadian clock gene to be cloned [8]. The expression pattern of *CCA1* has a circadian rhythm, peaking at dawn [8]. LHY is highly similar to CCA1 in sequence and function [9]. Constitutive expression of *CCA1* or *LHY* causes arrhythmicity and late flowering, whereas both single mutants (*cca1* or *lhy*) and double mutants (*cca1 lhy*) exhibit shortened rhythmic periods and early flowering [10]. CCA1 and LHY can function as transcription factors in the circadian rhythm process. As transcription inhibitors, CCA1 and LHY negatively regulate the expression of *GI* (*GIGANTEA*) and *TOC1* (*TIMING OF CAB EXPRESSION 1*); while as transcription activators, CCA1 and LHY promote the expression of *PRR7* (*Pseudo-Response Regulator*) and *PRR9* [11]. In addition, CCA1 and LHY play vital roles in flowering time. Flowering marks the transition from vegetative growth to reproductive growth, and the appropriate flowering time is crucial for reproduction [12]. It is well known that plants sense photoperiod changes and integrate light signals into the circadian clock to initiate or inhibit flowering. CCA1 and LHY are involved in regulating photoperiodic flowering by modulating GI-CO-FT pathways [13]. Moreover, many studies on the orthologs of CCA1 and LHY have also been implemented in other species, such as rice [14], soybean [15], maize [16], and barley [17].

Members of the MYB transcription factor superfamily play critical roles in plant development. MYB proteins are classified into four subfamilies according to the number of adjacent MYB repeats (R) [18]. Among them, CCA1 and LHY belong to the REVEILLE group of the MYB-related (1R-MYB) subfamily [19, 20]. Nine other proteins in *A. thaliana* also belong to this group, including RVE1-RVE8 and RVE7-like, all of which share the consensus sequence SHAQK(Y/F) F [21]. Except for RVE5 and RVE6, the expression of the remaining members is clock-regulated in seedlings [19]. Although CCA1 and LHY are essential regulators of the circadian clock, some other members of the 1R-MYB subfamily are also involved in this regulatory process. For example, RVE8 can directly promote the expression of *GI* and *TOC1* by binding with the evening element (EE), as is the case for the repressors CCA1 and LHY [19, 22]. Similarly, RVE4 and RVE6 can also bind with the EE and might be functionally redundant with RVE 8[23]. Moreover, increasing evidence indicates that RVE proteins play important roles in other biological processes. RVE1 regulates the auxin biosynthetic gene to connect the circadian clock and auxin pathways [24]. RVE8 modulates anthocyanin biosynthesis by promoting anthocyanin gene expression around dawn [25]. RVE4 and RVE8 function as essential transcription factors to regulate plant thermotolerance [26]. CCA1, LHY, RVE4 and RVE8 can trigger cold-inducible gene expression [27]. To date, RVE proteins have also been reported in other species. *GmMYB133,* as an *RVE* homologous gene in soybean, is involved in isoflavonoid biosynthesis [28]. Transient overexpression of *PbRVE1b* improves anthocyanin content in *P. bretschneideri* fruit skin [29]. Ectopic overexpression of *SgRVE6* in tobacco enhances cold tolerance [30]. Therefore, these results support that RVE proteins are significant for plant development.

Pear (*Pyrus*) is one of the representative fruits of Rosaceae species that is widely cultivated worldwide. Proper flowering and robust plant growth guarantee fruit quantity and quality. Although RVE proteins have been well studied in *A. thaliana*, the characterization of RVEs in Rosaceae species is limited. In this study, we identified 51 *RVE* genes from *P. bretschneideri* and six other Rosaceae species using published whole genome information. The phylogenetic relationship, chromosomal location, and evolutionary history were analyzed. Furthermore, we explored the protein motifs, gene structures and expression patterns of *PbRVEs*. Specifically, we demonstrate the function of PbLHY in repressing flowering time by ectopic transgenic analysis in *A. thaliana*. The results of this work contribute to further determining the functions of RVEs in Rosaceae species.

## Results

### Identification and phylogenetic analysis of *RVE* genes in seven Rosaceae species

**Fig. 1:**
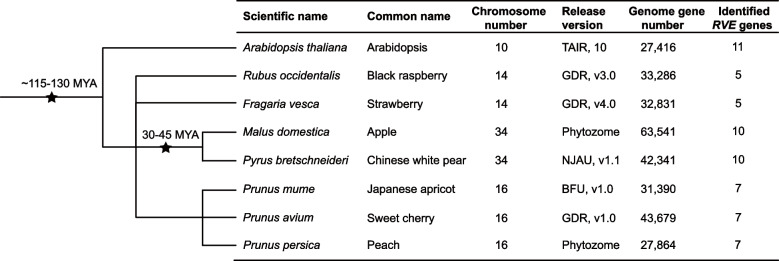
The evolutionary relationships, genome information, and the number of *RVE* genes in *Arabidopsis* and seven Rosaceae species. The black stars and the corresponding numbers above indicate the divergent events and the occurrence time, respectively. The first event (~ 115-130 MYA) was the ancient WGD event that occurred between *A. thaliana* and Rosaceae species, and the second event (30-45 MYA) was a recent WGD event that occurred before *Malus domestica* and *Pyrus bretschneideri*. MYA: millions of years ago

To obtain the members of RVE families in seven Rosaceae species genome, including *Pyrus bretschneideri*, *Malus domestica*, *Prunus mume*, *Prunus avium*, *Prunus persica*, *Rubus occidentalis*, *Fragaria vesca*, an HMMER-BLASTP strategy was first used to search for the homologs with HMM profile (PF000249) and RVE protein sequences of *A. thaliana* were used as queries. RVE proteins have a single conserved MYB domain with a SHAQK(F/Y) F sequence. The candidate proteins were further verified by NCBI Batch CD-Search and multiple sequence alignment. In total, fifty-one RVE members were identified in this study: five in *R. occidentalis*, five in *F. vesca*, ten in *M. domestica*, ten in *P. bretschneideri*, seven in *P. mume*, seven in *P. avium*, and seven in *P. persica* (Fig. 1 and Table S1).

Pbr025890.1 with an incomplete MYB domain was identified as a homolog of AtCCA1 and AtLHY by BLASTP in the Chinese white pear genome [31]. However, the protein sequence of XP_009335251 (the corresponding ID of Pbr025890.1 in the NCBI database) with a complete MYB domain showed 38.18% and 45.24% sequence similarity with AtCCA1 and AtLHY, respectively (Fig. S1). Therefore, we retained this protein as a member of the PbRVE family and named it PbLHY. The protein sequence downloaded from NCBI was used for subsequent analysis. In addition, other *PbRVE* genes were named according to their homologous genes in *A. thaliana*.

**Fig. 2:**
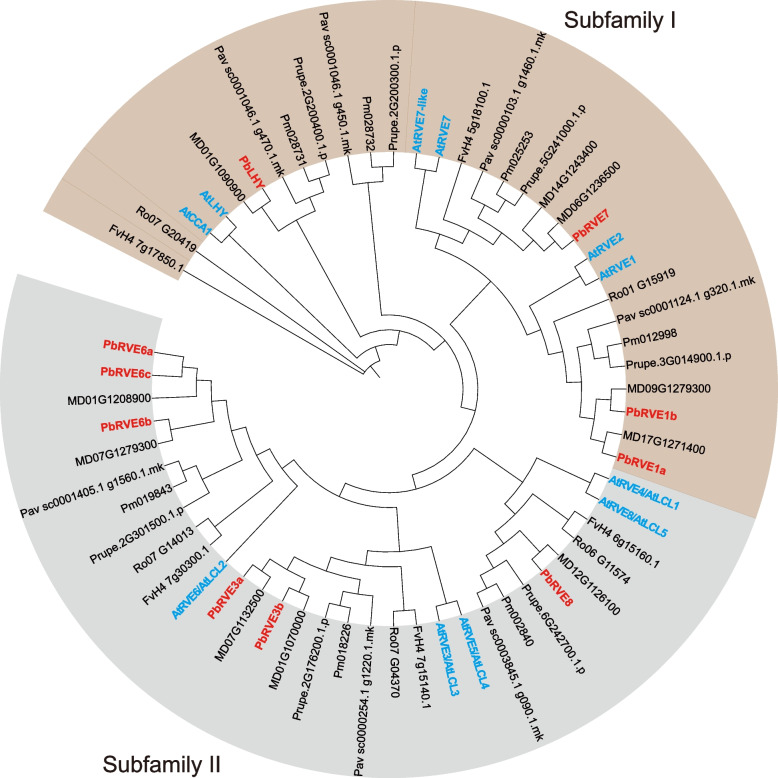
Phylogenetic tree of RVE proteins in *Arabidopsis* and seven Rosaceae species. The blocks in brown and gray represent members in subfamilies I and II, respectively. The RVE proteins in *Pyrus bretschneideri* are labeled in red, and the RVE proteins in *A. thaliana* are labeled in blue

To investigate the evolutionary relationships of the RVE family between Rosaceae species and *A. thaliana*, we constructed a maximum-likelihood phylogenetic tree using putative protein sequences. Based on the topology of the phylogenetic tree and the classification of RVEs in *A. thaliana*, all proteins from eight species were clearly divided into two groups, namely, subfamily I and subfamily II (Fig. 2). PbRVEs containing an additional LCL (LHY/CCA1-Like) domain were assigned to subfamily II, whereas PbRVEs without this domain were assigned to subfamily I (Fig. S2). The phylogenetic tree showed that the majority of Rosaceae RVE members were clustered together with *A. thaliana* RVE proteins. However, the number of *RVE* genes in *M. domestica* and *P. bretschneideri* was greater than that in the other five species, which is likely because of the lineage-specific whole-genome duplication (WGD) event (30-45 MYA) that occurred before the split of *Malus* and *Pyrus* lineages (Fig. 1).

### Chromosomal location and evolution events of *RVE* genes

**Fig. 3:**
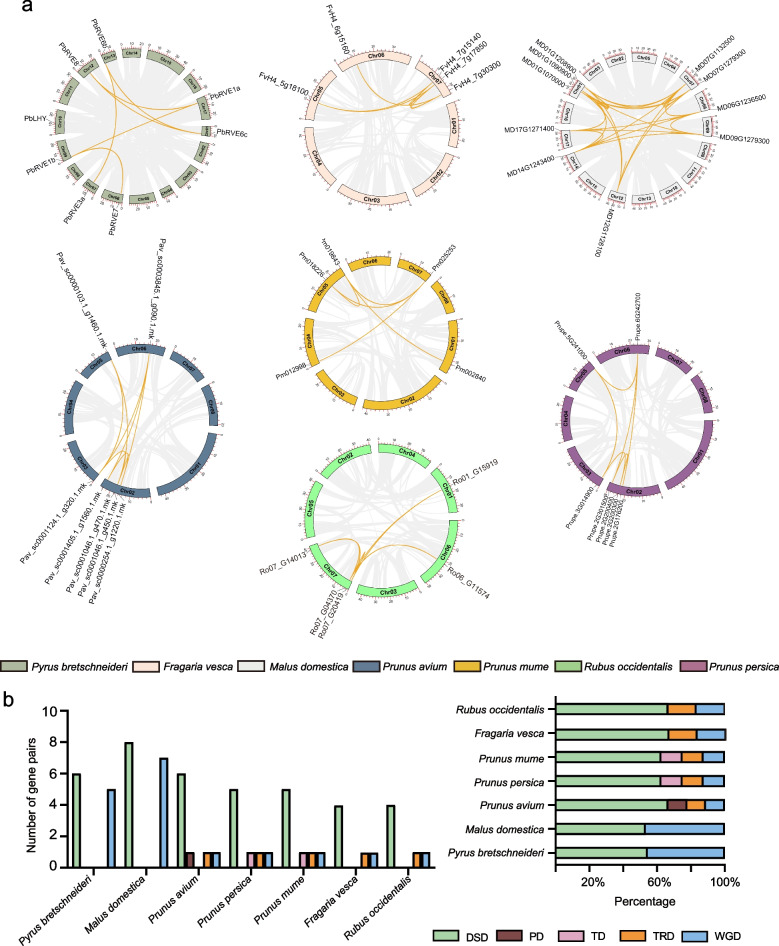
Gene intragenomic collinearity and duplication events analysis of the RVE family in Rosaceae species. (a) Localization and intragenomic collinearity of *RVE* genes. The seven colored circles represent the chromosomes of the seven Rosaceae species. The approximate positions of *RVE* genes are marked with short black lines on the circles. The yellow lines represent gene pairs with syntenic relationships. Genes located on the scaffolds are not shown. (b) Statistics for different duplication events in seven species, including *Pyrus bretschneideri*, *Malus domestica*, *Prunus avium*, *Prunus persica*, *Prunus mume*, *Fragaria vesca* and *Rubus occidentalis*. The five colored rectangles represent different events, DSD (green), PD (brown), TD (pink), TRD (orange), and WGD (blue). The Y-axis of the left column chart represents the number of duplicated gene pairs in each event. The X-axis of the right column chart represents the percentage of the duplicated gene pairs in each event

To analyze the genomic distribution characteristics of *RVE* members, the locations of 51 *RVE* genes in seven Rosaceae species were detected (Table S1). Eight *PbRVEs* were mapped onto eight out of 17 *P. bretschneideri* chromosomes, and another two *PbRVEs* (*PbRVE3b* and *PbRVE6a*) were located on the scaffolds. We also found that five *FvRVEs* were located on three *F. vesca* chromosomes, five *PmRVEs* on four *P. mume* chromosomes, seven *PaRVEs* on four *P. avium* chromosomes, ten *MdRVEs* on seven *M. domestica* chromosomes, seven *PpRVEs* on four *P. persica* chromosomes, and five *RoRVEs* on three *R. occidentalis* chromosomes (Fig. 3a). Notably, *RVE* genes of different species were unevenly distributed across the chromosomes.

Gene duplication events contribute to driving the expansion of gene families and generating new functions. We investigated the duplication events of *RVE* genes using the DupGen_finder pipeline. Five modes, including WGD, transposed duplication (TRD), tandem duplication (TD), proximal duplication (PD), and dispersed duplication (DSD), were detected with different contributions to the *RVE* family in seven Rosaceae species (Fig. 3b and Tables S2, S3). DSD was the dominant event, accounting for 60% of the 63 *RVE* duplicated gene pairs. In *P. bretschneideri* and *M. domestica*, *RVE* gene pairs were only derived from DSD and WGD, which were almost evenly distributed. Interestingly, the numbers and percentages of *RVE* genes originating from DSD, TRD and WGD were equal in *F. vesca* and *R. occidentalis*; the numbers and percentages of *RVE* genes originating from DSD, TRD, TD and WGD were equal in *P. mume* and *P. persica*.

The Ks value is used to estimate the evolutionary time of duplication events, such as the recent WGD (Ks ~ 0.15-0.3) and the ancient WGD (Ks ~ 1.5-1.8) of the *P. bretschneideri* genome [31]. In our study, the Ks value of *P. bretschneideri RVE* gene pairs ranged from 0.023 to 2.585 (Table S3). Among them, *PbRVE6c* and *PbRVE6a* had the lowest Ks value, while *PbRVE3b* and *PbRVE7* had the highest Ks value. In addition, the Ks values of the other six Rosaceae species varied in different ranges. Next, we calculated Ka/Ks ratios to estimate the selection pressure driving the evolution of the *RVE* family. The ratios of all *RVE* gene pairs were lower than one, indicating that purifying selection was the main evolutionary driving force on *RVE* families in Rosaceae species (Table S3).

### Protein and gene feature analysis of RVE members in *P. bretschneideri*

**Fig. 4:**
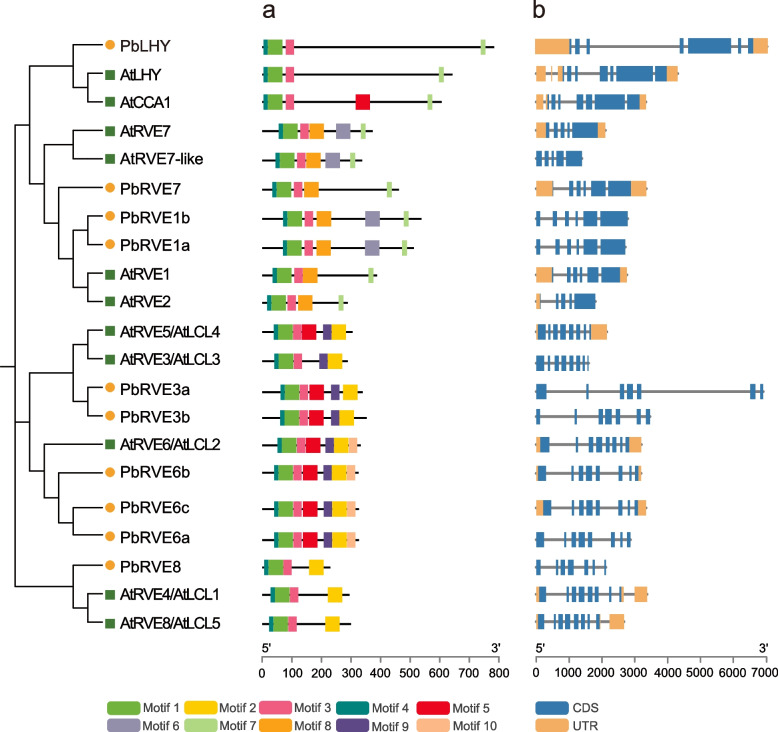
Analysis of the conserved motifs and gene structures of *RVE* genes in *Arabidopsis* and *Pyrus bretschneideri*. (a) Conserved motifs of PbRVE and AtRVE proteins. Ten conserved motifs were predicted by MEME. Different colored boxes represent different motifs. The proteins are arranged according to the phylogenetic tree. PbRVE proteins are labeled with yellow circles, and AtRVE proteins are labeled with green squares. (b) Exon–intron structure of *PbRVE* and *AtRVE* genes. Blue boxes, orange boxes, and gray lines represent exons, UTRs and introns, respectively

To further explore the conservation and diversity of PbRVE family members, we analyzed the features of their protein and gene sequences. The protein length of PbRVEs ranged from 226 to 777 amino acids (Table S1). Ten putative motifs were identified by the MEME tool (Fig. 4a and Fig. S3). All members of the PbRVE family contained motifs 1, 3, and 4, which represented the single conserved MYB domains. Motif 2, characterized as the LCL domain, was detected in all members of subfamily II. Motif 7 was identified only in all members of subfamily I. Motifs 6 and 8 were unique in some members of subfamily I, while motifs 9 and 10 appeared only in some members of subfamily II. These results supported the phylogenetic relationship and classification of the RVE family, and the specific motif patterns of different branches suggested that there might be functional divergence in these RVE members.

To analyze the structural characteristics of *RVE* genes, exon–intron compositions were investigated by the Gene Structure Display Server (GSDS) tool (Fig. 4b). The numbers of exons/introns were generally similar between *RVE* genes in *P. bretschneideri* and *A. thaliana*. Moreover, members in the same subfamilies shared relatively conserved gene structures. For example, the numbers of exons ranged from five to seven in subfamily I, whereas they ranged from seven to eight in subfamily II.

### *Cis*-element analysis of putative *PbRVE* promoters

To elucidate the potential regulatory functions of *PbRVE* genes, 1500 bp promoter regions (upstream of the start codons) were obtained and analyzed by the PlantCARE tool. The promoter region of each gene was enriched with multiple *cis*-elements. Next, we focused on the *cis*-elements related to plant hormones, low temperature and light response in the promoters of *PbRVEs* (Table 1). All *PbRVE* genes except *PbLHY* might be involved in the abscisic acid response. Among them, *PbRVE1a*, *PbRVE3a* and *PbRVE6a* contained more than five ABRE *cis*-elements. Meanwhile, *cis*-elements related to auxin, gibberellin and MeJA responses were identified in the majority of *PbRVEs*. Notably, the promoters of eight *PbRVEs* contained LTR *cis*-elements, indicating that *PbRVE* genes might play roles in the low-temperature response. As expected, light-responsive elements were highly conserved in the promoter of *PbRVEs*. Specifically, only *PbLHY* contained one MRE element (MYB binding site involved in light responsiveness), and the rest of the *PbRVEs* contained different numbers of G-box elements.

**Table 1:** Types and numbers of responsive elements in the promoter regions of *PbRVE* genes

Function	Auxin		Abscisic acid	Gibberellin		MeJA	Ethylene	Salicylic acid	Low-temperature	Light		
Element	AuxRR	TGA	ABRE	P-box	GARE	CGTCA	ERE	TCA	LTR	GATA	G-box	MRE
PbLHY	1	0	0	0	0	1	0	1	1	0	0	1
PbRVE1a	1	1	5	0	0	3	0	0	2	1	5	0
PbRVE1b	0	0	1	0	0	0	0	2	1	0	2	0
PbRVE3a	0	0	12	1	0	3	0	0	1	1	12	0
PbRVE3b	0	1	3	0	0	0	0	0	0	0	3	0
PbRVE6a	0	1	5	1	1	2	0	0	1	1	5	0
PbRVE6b	1	0	3	2	1	1	1	0	1	0	3	0
PbRVE6c	1	1	2	1	0	0	2	1	1	1	2	0
PbRVE7	0	0	3	0	0	1	2	0	1	0	3	0
PbRVE8	0	0	2	0	1	1	0	0	0	0	3	0

### Expression analysis of *RVE* genes in *P. bretschneideri*

**Fig. 5:**
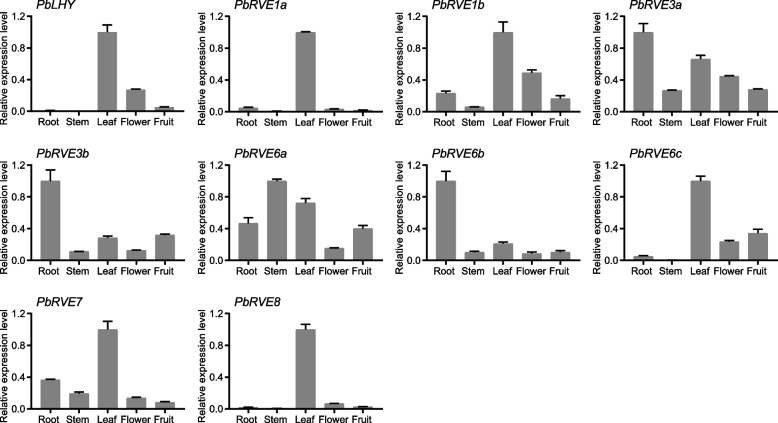
Tissue-specific expression patterns of *PbRVE* genes. The expression patterns of ten *PbRVEs* were detected in the roots, stems, leaves, flowers, and fruits of *Pyrus bretschneideri* trees using qRT–PCR. *PbUBQ* was used as an internal control. The highest expression level of each gene was set to 1 for standardized calculation. Error bars represent the standard error

To explore the potential functions of *PbRVE* genes, we first examined their specific expression levels in five tissues using a quantitative real-time PCR (qRT–PCR) assay. *PbRVE* genes displayed diverse expression patterns in different tissues (Fig. 5). Most members were highly expressed in leaves. For example, *PbLHY*, *PbRVE1a*, and *PbRVE8*, except for a small amount of transcription in flowers, were mainly expressed in leaves, suggesting that they may play critical roles in leaves. In addition, *PbRVE3a*, *PbRVE3b* and *PbRVE6b* shared similar expression profiles with higher expression levels in roots than in other organs. *PbRVE6a* was detected in all five tissues, and the expression level was the highest in the stem.

**Fig. 6:**
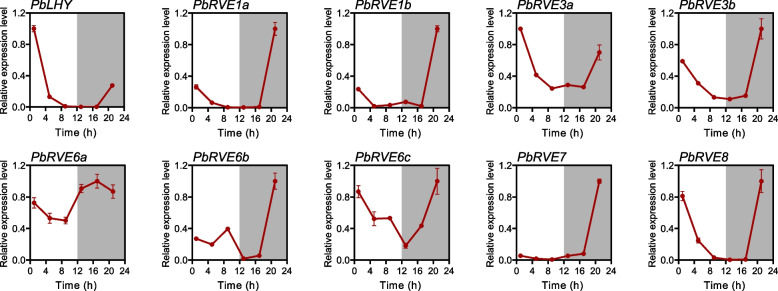
Diurnal expression patterns of *PbRVE* genes under 12-h light/12-h dark conditions. The time of the light beginning to turn on was defined as zeitgeber time zero (ZT 0). Starting from ZT1, leaves were harvested every 4 h for a total of 6 times throughout the whole day. White and black areas indicate light and darkness, respectively. *PbUBQ* was used as an internal control. The highest expression level of each gene was set to 1 for standardized calculation. Error bars represent the standard error

**Fig. 7:**
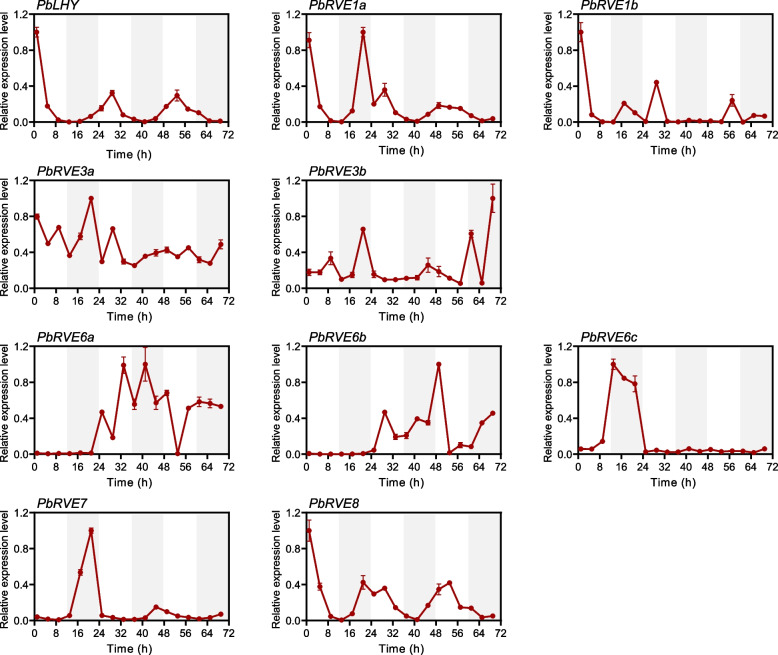
Circadian expression patterns of *PbRVE* genes under constant light conditions. The time of the light beginning to turn on was defined as zeitgeber time zero (ZT 0). Starting from ZT1, leaves were harvested every 4 h for a total of 18 times over three days. White and gray areas indicate subjective day and subjective night, respectively. *PbUBQ* was used as an internal control. The highest expression level of each gene was set to 1 for standardized calculation. Error bars represent the standard error

Previous studies have well proven that partial RVEs (CCA1, LHY and RVE8) are key circadian clock components in *A. thaliana* [2]. The expression of these genes was clock-regulated and showed rhythmic oscillations in seedlings [19]. To investigate the responses of *PbRVE* genes to the diurnal cycle and circadian clock, we detected the expression changes of *PbRVE* genes in *P. bretschneideri* leaves under 12-h light/12-h dark conditions (Fig. 6) and constant light conditions (Fig. 7). Under light/dark cycles, all *PbRVE* genes exhibited diurnal rhythms that could be divided into three types (Fig. 6). The expression of *PbLHY* and *PbRVE3a* peaked at dawn (zeitgeber time 1; ZT 1), then dropped steeply during the day and increased slightly before dawn. In *P. bretschneideri*, *PbLHY,* as the only homologous gene of *AtCCA1* and *AtLHY,* shared the same expression pattern as the two genes in *A. thaliana*. In contrast, several *PbRVE* genes showed the highest expression levels before dawn (ZT 21), such as *PbRVE1a* and *PbRVE1b*. *PbRVE6a* showed a completely different pattern, characterized by a peak in the evening (ZT 17).

Circadian-controlled rhythms still persist with robust rhythms when a plant is transferred to a constant light environment (free-running conditions) [3]. In our study, after growing in 12-h light/12-h dark conditions for a time, *P. bretschneideri* seedlings were treated with constant light for three days, and the expression patterns of *PbRVE* genes were detected. The expression of *PbLHY*, *PbRVE1a*, *PbRVE7* and *PbRVE8* was clock-regulated (Fig. 7). The peak time on the first day was similar to that under light/dark cycles, occurring near dawn. As time progressed (the second and third days), they maintained the circadian rhythm, but the period lengthened and the expression amplitude decreased. In conclusion, *PbLHY*, *PbRVE1a*, *PbRVE7* and *PbRVE8* showed diurnal and circadian rhythms, implying that they might be involved in the circadian clock system.

### Subcellular localization of PbLHY protein

**Fig. 8:**
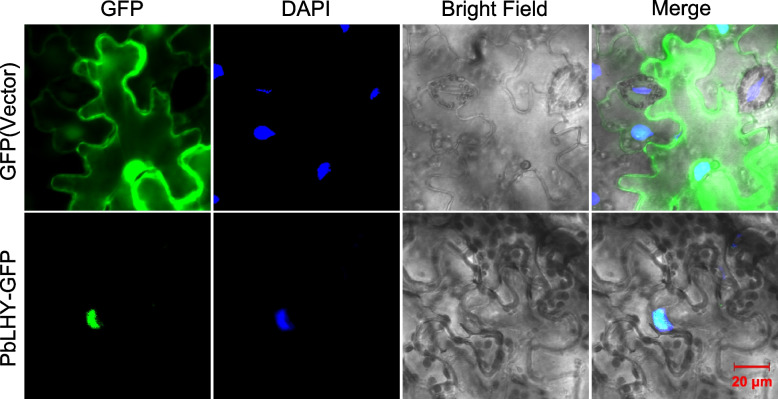
Subcellular localization of the PbLHY-GFP in tobacco leaf epidermal cells. The images were obtained using laser scanning confocal microscopy. Green represents GFP fluorescence. Blue represents DAPI staining as the nuclear marker. The empty vector of *35S*: GFP was used as a control. Bar = 20 μm

In *A. thaliana*, CCA1 and LHY function as transcription factors and are localized in the nucleus [20]. To further examine the role of PbLHY, subcellular localization was performed using a construct with the full coding region of *PbLHY* fused to green fluorescent protein (GFP). When the construct was transiently transformed into leaf epidermal cells of tobacco, a fluorescent signal of PbLHY-GFP was observed in the nucleus (Fig. 8), indicating that PbLHY is a nuclear-localized protein.

### Overexpression of *PbLHY* delayed flowering in *A. thaliana*

**Fig. 9:**
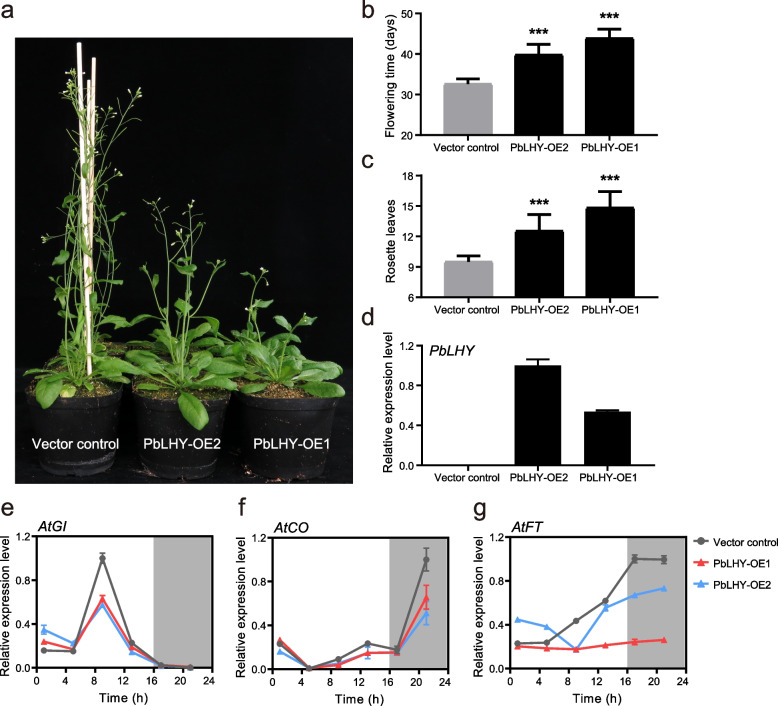
Overexpression of *PbLHY* delayed flowering in *Arabidopsis*. (a) Flowering phenotypes of representative control plants and *PbLHY*-OE lines grown under long-day conditions for six weeks. (b) and (c) The number of days and rosette leaves were recorded in control and *PbLHY*-OE lines when the first flower was visible. *** *p* < 0.001 vs. vector control by *t test*. (d) Expression of *PbLHY* in control and *PbLHY*-OE lines. (e-g) Expression of *AtGI*, *AtCO*, and *AtFT* in control and *PbLHY*-OE lines. Transcript levels were measured by qRT–PCR assay in 12-day-old seedlings under long-day conditions and normalized to *AtACT*. The highest expression level of each gene was set to 1 for standardized calculation. Error bars represent the standard error. White and black areas indicate light and darkness, respectively

Considering the high similarity between PbLHY and *A. thaliana* CCA1/LHY in sequence characteristics and expression patterns, we selected PbLHY for further functional study on regulating flowering time. In *A. thaliana*, *lhy-1* mutation, which was an *AtLHY* overexpression line, caused late flowering under long-day conditions [10]. Due to the lack of a dependable transformation system and the extremely long time from seedling to florescence in *P. bretschneideri*, we transformed PbLHY into *A. thaliana* to observe the flowering phenotype. *PbLHY* showed significantly high expression levels in two independent transgenic lines but was not detected in the vector control plants (transformed with an empty vector) (Fig. 9d). Under long-day (16 h light/8 h dark) conditions, we counted the number of days and rosette leaves when the first flower bud opened. *PbLHY* overexpression (OE) lines exhibited an obviously delayed flowering phenotype (Fig. 9a). The average flowering time of the control plants was 32.58 days, while that of the *PbLHY*-OE lines was 39.78 days and 43.90 days (Fig. 9b). The number of rosette leaves in *PbLHY*-OE lines increased significantly compared with that in control plants (Fig. 9c), suggesting that *PbLHY* might play a conserved role in suppressing flowering.

Furthermore, we assessed the effect of PbLHY on flowering time-related genes in *A. thaliana*, including *AtGI*, *AtCO*, and *AtFT*. Although the expression waveforms of *AtGI* and *AtCO* in *PbLHY*-OE lines were similar to those in control plants, the expression levels were changed at different time points. For example, *AtGI* transcripts were significantly repressed at ZT9 (Fig. 9e), and *AtCO* transcripts were reduced at ZT13 and ZT21 (Fig. 9f) in both *PbLHY*-OE lines. The expression levels of *AtFT* were also decreased in *PbLHY*-OE lines at several time points during the day (Fig. 9g). The results indicated that *PbLHY* might delay flowering and accompanied by repression of these key factors.

## Discussion

The life cycle of flowering plants includes vegetative growth and reproductive growth. Flowering is a symbolic process of the plant transition from vegetative development to reproductive growth. Several pathways and numerous genes have been implicated in regulating flowering, among which circadian clock genes play essential roles [32]. Most plants have evolved an endogenous circadian clock system to adapt to the periodic changes caused by the Earth’s rotation [33]. In addition to flowering, circadian clock genes also influence multiple physiological processes, such as plant growth, hormone signaling, metabolism and stress response. *CCA1* and *LHY* are considered to be the most central genes for clock functions [2]. However, *CCA1* and *LHY* in *P. bretschneideri* have not been previously reported. In this study, we performed an evolutionary analysis of the RVE family in seven Rosaceae species and found that *PbLHY* delay flowering time in *A.thaliana*. This result suggested that *PbLHY* may act as flowering time repressor.

Over the past decades, the MYB superfamily has been extensively studied. Genome-wide identification of MYB family members has been carried out in several species, such as *A. thaliana* [18], apple [34], pear [35, 36], peach [37], and strawberry [38]. Based on the number of repeats, MYB proteins are classified into four subfamilies: MYB-related (which contain a single MYB-like domain), R2R3, R1R2R3, and 4R [18]. It is considered that the MYB family has been selectively extended in plants, especially through the R2R3 subfamily. Therefore, previous studies focused more on the analysis of R2R3-MYB [39]. However, increasing evidence has shown that MYB-related members also play important roles in multiple stages of plant development, particularly the RVE group to which CCA1 and LHY belong [30, 40–42]. Because the RVE group is a small branch of the MYB superfamily, it is necessary to conduct detailed analysis to avoid missing important members. Although several studies have identified MYB family members in pear [29, 35, 36], we still obtained new members through strict screening. We identified ten *RVE* genes in *P. bretschneideri*, among which *PbLHY*, *PbRVE6b* and *PbRVE6c* were reported for the first time.

In *A. thaliana*, the RVE family, including 11 proteins, is divided into two subfamilies according to the MYB and LCL domains [22]. All RVE proteins in the seven Rosaceae species and *A. thaliana* were clustered into two groups in our results, but the number of RVE members in different species was obviously distinct. *P. bretschneideri* (10) and *M. domestica* (10) had a similar number of RVE members to *A. thaliana*, while *F. vesca* (5) and *R. occidentalis* (5) had only half the number of *A. thaliana*. In addition, *P. mume*, *P. avium*, and *P. persica* belong to Prunoideae with the same number of RVE proteins, seven. *P. bretschneideri* and *M. domestica* belong to Maloideae with the same number of RVE proteins, ten. The phenomenon also occurred with *F. vesca* and *R. occidentalis*, both of which belong to Rosoideae [43]. It has been reported that a recent WGD has occurred in Maloideae [31]. Therefore, WGD might be one of the primary driving forces for the expansion of the RVE family in *P. bretschneideri* and *M. domestica*. To further investigate the evolutionary process of *RVE* genes in Rosaceae, we analyzed gene duplication events in each species. Overall, DSD was the main duplication event of *RVE* genes in Rosaceae. DSD is ubiquitous in plant genomes and occurs in unpredictable and random patterns, producing two gene copies that are neither adjacent nor collinear [44]. The events of the *RVE* gene family in *P. bretschneideri* and *M. domestica* were only a WGD in addition to DSD, which clearly indicated that WGD was another main driving force for the evolution and expansion of these two RVE families. Meanwhile, the types of duplication events were similar between Rosaceae species that were more closely related. Since duplication events contribute greatly to the evolution and function of genes [45], it is worthwhile to understand RVE family members by analyzing gene duplication events.

In *A. thaliana*, *RVE* genes play important roles in controlling the circadian clock, photoperiod flowering, anthocyanin biosynthesis, hormone signaling, and stress responses [10, 24–26]. In our study, the expression pattern of *PbRVEs* varied in different tissues, indicating that *PbRVEs* may have potential functions in plant development. For example, *PbLHY* and *PbRVE8* were highly expressed in leaves, which was consistent with previous reports of homologs in poplar [46], rice [47], and mungbean [48]. Leaves are the main sites for sensing light signals and measuring time [49], and the high expression of these genes in leaves suggested that they may participate in the photoperiod pathway of *P. bretschneideri*. Meanwhile, we examined the expression of *PbRVE* genes under light/dark cycles and constant light conditions to explore their expression characteristics in leaves. *PbLHY*, *PbRVE1a*, *PbRVE7*, and *PbRVE8* exhibited both typical diurnal and circadian rhythms, peaking around dawn. These genes not only have highly conserved structures and motifs but also have the same expression patterns as their homologs in *A. thaliana* [10, 19], suggesting that they may play similar roles in circadian clock regulation in *P. bretschneideri*. Additionally, transcripts of many *RVE* genes showed relatively high levels in roots, such as *PbRVE3b* and *PbREVE6b*. The function of *RVE* genes in roots has rarely been reported before. Recently, transcriptome analysis of *Juglans regia* and *Medicago truncatula* found that *RVE* genes may be involved in the nitrogen metabolic pathway [50, 51]. Moreover, *RVE* genes had diverse expression patterns in different developmental stages of jujube fruit [52], and *PbRVE1b* promoted anthocyanin accumulation in *P. bretschneideri* peel [29]. In our study, *PbRVE3b* and *PbRVE6c* were also highly expressed in fruit, implying that they may be involved in *P. bretschneideri* fruit development.

PbLHY, which was the only homolog of CCA1 and LHY in *P. bretschneideri*, was filtered in the *P. bretschneideri* genome due to the lack of conserved residues. However, the full-length *PbLHY* gene containing the complete conserved domain was cloned. Our results complemented the conservation of circadian clock genes in plants [33]. Furthermore, the nuclear localization of PbLHY indicated that it may function as a transcription factor similar to CCA1 and LHY in *A. thaliana* [20]. In long-day representative plants such as *A. thaliana*, the dynamic interaction of photoperiodic flowering is largely controlled by the level of GI-CO-FT module activity [13]. In short-day plants, such as rice, OsGI-Hd1-Hd3a is the parallel module [49]. In both of these annual model plants, AtCCA1/AtLHY and OsLHY affect flowering mainly through that conserved pathway [13, 47]. Overexpressing *PbLHY* could delay flowering in transformed *A. thaliana*. The expression of *GI*, *CO*, and *FT* was repressed at different time points in *PbLHY*-OE lines. Thus, we proposed that *PbLHY* was the candidate gene as a circadian clock component and regulated flowering in *P. bretschneideri*. Further studies will focus on confirming this hypothesis and exploring more functions of the *PbRVE* genes.

## Conclusions

In this study, we identified 51 *RVE* genes in seven Rosaceae species and investigated their evolutionary characteristics, such as phylogenetic relationship, chromosomal locations, and duplication events. Then, a systematical analysis was carried out to study *RVE* members in *P. bretschneideri*. Based on the analysis of gene structures, conserved motifs, and phylogenetic tree, *PbRVE* genes were clustered into two subfamilies. The expression levels of ten *PbRVE* genes varied among *P. bretschneideri* tissues. Under 12-h light/12-h dark conditions, ten *PbRVE* genes exhibited diurnal rhythms; among them, four *PbRVE* genes also exhibited rhythms under constant light conditions. In addition, overexpression of *PbLHY* could delay flowering time in transgenic *A. thaliana* plants. These results provide evidence for the functional verification of the *P. bretschneideri* clock genes and represent a foundation for further functional characterization of RVE genes in Rosaceae.

## Methods

### Collection of genome data and identification of *RVE* genes

The genome sequence of Chinese white pear (*Pyrus bretschneideri*) was downloaded from the Pear Genome Project (http://gigadb.org/dataset/100083) [31]. The genome sequences of *M. domestica* [53] and *P. persica* [54] were downloaded from Phytozome (https://phytozome-next.jgi.doe.gov). The genome sequences of *R. occidentalis* [55], *F. vesca* [56], *P. avium* [57], and *P. mume* [58] were downloaded from the Genome Database for Rosaceae (GDR; https://www.rosaceae.org/). The genome sequence of *A. thaliana* was downloaded from the *Arabidopsis* Information Resource (TAIR; http://www.Arabidopsis.org/) [59]. Protein sequences of 11 AtRVEs were used as queries to perform BLASTP searches against the seven Rosaceae genome databases (*P* value <1e^− 10^). Protein sequences of candidate RVEs in the seven Rosaceae species were analyzed by Pfam and SMART to verify the MYB domain. The sequences without the MYB domain were deleted.

### Phylogenetic analysis and multiple sequence alignment

The RVE protein sequences of the seven Rosaceae species and *A. thaliana* were aligned by MAFFT v7.0 (https://mafft.cbrc.jp/alignment/software/) [60]. The results of multiple sequence alignment were visualized by Jalview (http://www.jalview.org/). The maximum likelihood phylogenetic tree of the RVE family was created by iqtree (http://www.iqtree.org/) (bootstrap =1000) and visualized by iTOL (https://itol.embl.de/).

### Collinearity analysis and Ka and Ks calculation

Collinearity analysis among the seven Rosaceae genomes was conducted locally using a method similar to that developed for the Plant Genome Duplication Database (http://chibba.agtec.uga.edu/duplication/) [61]. Candidate homologous *RVE* gene pairs were identified using DIAMOND software with the parameters --bin 2 --max-target-seqs 5 --e-value 1e^− 10^. Then, the *RVE* gene pairs and their chromosome location information were fed into MCScanX software to identify the collinear chains with default parameters. Genes located on the scaffolds were removed. The result was visualized by TBtools [62]. Duplication events in the *RVE* gene family were identified using the DupGen_finder pipeline [45]. The nonsynonymous substitution rates (Ka) and the synonymous substitution rates (Ks) of homologous *RVE* gene pairs in each Rosaceae species were calculated by KaKs_Calculator 2.0 using the Nei-Gojobori method [63].

### Conserved motifs, gene structures and *cis*-regulatory element analysis

Conserved motifs of RVE proteins in the seven Rosaceae species were analyzed by MEME tool v5.0.5 (http://meme-suite.org/tools/meme) [64] with the following parameters: maximum numbers of different motifs--10. Gene structures of *RVEs* in the seven Rosaceae species were plotted by the Gene Structure Display Server (http://gsds.cbi.pku.edu.cn/) [65]. These results were visualized by TBtools. The 1500 bp sequences ahead of the initiation codon (ATG) of *PbRVE* genes were downloaded from the Pear Genome Project [31]. *Cis*-acting regulatory elements of *PbRVE* genes were obtained using the PlantCARE database (http://bioinformatics.psb.ugent.be/webtools/plantcare/html/) [66].

### Plant materials and growth conditions

For tissue-specific expression analysis, all materials were harvested from ten-year-old *Pyrus bretschneideri* Rehd. cultivar ‘Dangshansuli’ trees grown in the Nanjing Agricultural University Experimental Field, China. The harvested seeds from ‘Dangshansuli’ trees were vernalized at 4 °C in sand for 2 months. Then, the rooted seedlings were selected and transferred to a room with settings of 22 °C, 100 μmol m^− 2^ s^− 1^ light, and 65% relative humidity. All seedlings had grown under 12-h light/12-h dark conditions for 30 days. The time of the light beginning to turn on was defined as zeitgeber time zero (ZT 0). For diurnal rhythm analysis, leaves of seedlings were collected every 4 h for one day. For circadian rhythm analysis, seedlings were released to constant light, and the leaves were collected every 4 h for 3 days. The leaves of three seedlings with uniform growth were randomly selected as a mixed sample at the designated time point. Three independent biological repeats were collected for the experiments. All samples were frozen immediately in liquid nitrogen and stored at − 80 °C.

*A. thaliana* plants were grown under long-day conditions (16-h light/8-h dark) in the controlled room with settings of 22 °C, 100 μmol m^− 2^ s^− 1^ light, and 65% relative humidity. Seeds of transgenic *A. thaliana* plants were sterilized and screened on Murashige and Skoog (MS) medium with 20 mg L^− 1^ hygromycin. Flowering phenotypes were observed in the T3 generation. For gene expression analysis, seeds were grown in MS medium for 12 days, and then the seedlings were collected every 4 h for one day.

### RNA extraction and quantitative real-time PCR (qRT–PCR)

Total RNA was extracted from frozen tissues using a Plant Total RNA Isolation Kit (FOREGENE, Chengdu, China) specifically designed for plants rich in polysaccharides and polyphenols. One microgram of total RNA was reverse transcribed in a 20 μl reaction volume using One-Step gDNA Removal and cDNA Synthesis SuperMix (TransGen Biotech, Beijing, China). Gene-specific primers of *PbRVEs* were designed by Primer Premier 5.0 software, and the specificity was verified by the Primer search program against the *P. bretschneideri* genome (Table S4). To test relative gene expression levels, qRT–PCR was performed using SYBR Green I Master Mix (Roche, Germany) in a Roche LightCycler 480 II. The reactions were prepared in a total volume of 20 μl containing 0.1 μl of cDNA, 5 μl of 0.5 μM gene-specific primer premix, 10 μl of 2 × SYBR Green Master Mix, and 4.9 μl of water. Data were calculated using the 2^-ΔCT^ method. *PbUBQ (POLYUBIQUITIN)* of *P. bretschneideri* or *AtACT (ACTIN)* of *A. thaliana* was used as the reference control to normalize the expression of the target genes. Three biological replicates and three technical replicates were used for each experiment. All qRT–PCR experiments followed MIQE guidelines [67].

### Subcellular localization and *A. thaliana* transformation

Full-length cDNA without the termination codon of *PbLHY* was amplified by PCR. The product was inserted into the pCAMBIA1300-35S: CDS-GFP vector [68] using the ClonExpress II One Step Cloning Kit (Vazyme Biotech, Nanjing, China). After sequencing, the recombinant plasmid and vector control plasmid were transformed into Agrobacterium competent GV3101 cells. For the subcellular localization assay, Agrobacterium-mediated transient expression in tobacco (*Nicotiana benthamiana*) leaves was performed according to a published protocol [69]. DAPI (Thermo Fisher Scientific, US) was used as a nuclear counterstain. The fluorescence in the transformed cells was imaged under a laser scanning confocal microscope LSM800 (Zeiss, Germany). For the *A. thaliana* transformation assay, the above Agrobacterium strains were transformed into *A. thaliana* (Columbia-0) plants using the floral dip method [70]. The *PbLHY* transgenic lines were confirmed by PCR with gene-specific and vector primers (Table S4). The transgenic lines of the T3 generation were used to observe the phenotypes and perform other analyses. Seeds of T3 generation were also sterilized and screened on MS medium with 20 mg L^− 1^ hygromycin. After 3 days of vernalization, the seeds were grown under long-day conditions mentioned above. The time of the seeds normally exposed to light was defined as the beginning day. And flowering days were counted from the beginning day to the first flower opening after bolting. At the same time, the number of rosette leaves was counted.

## Supplementary Information

**Additional file 1: Fig. S1.** (a) Coding sequence of *PbLHY*. (b) Alignment of PbLHY and AtCCA1/AtLHY protein sequences. The red box indicates the MYB domain. The blue backgrounds correspond to the percent identity of the multiple alignment. **Fig. S2.** Alignment of conserved domains from PbRVE proteins. (a) Alignment of MYB domains from ten PbRVEs and eleven AtRVEs. Red box indicates MYB domain, and blue box indicates SHAQK(Y/F) F sequence. (b) Alignment of LCL domains from subfamily II members. The red box indicates the MYB domain. The green box indicates the LCL domain. The blue backgrounds correspond to the percent identity of the multiple alignment. **Fig. S3.** The motif details of ten conserved domains identified in RVE proteins and the logos of these domains created using the MEME. Amino acids are expressed in the standard single letter code. The size of the letters at each position represents their frequency.

**Additional file 2: Table S1.** Characteristics of RVE family members in seven Rosaceae species. **Table S2.** Numbers of RVE gene pairs from different duplication events in seven Rosaceae species. **Table S3.** Ka and Ks calculation of duplicated RVE gene pairs in seven Rosaceae species. **Table S4.** The primers used in the assays.

## Data Availability

All data used in this study are included in this article and additional flies. All genome sequence used in this study are publicly available in: *A. thaliana* (http://www.Arabidopsis.org/), *P. bretschneideri* (http://gigadb.org/dataset/100083), *M. domestica* and *P. persica* (https://phytozome-next.jgi.doe.gov), *R. occidentalis*, *F. vesca*, *P. avium*, and *P. mume* (https://www.rosaceae.org/).

## Abbreviations

RVE: REVEILLE
CCA1: CIRCADIAN CLOCK ASSOCIATED 1
LHY: LATE ELONGATED HYPOCOTYL
GI: GIGANTEA
TOC1: TIMING OF CAB EXPRESSION 1
PRR7: Pseudo-Response Regulator 7
CO: CONSTANS
FT: FLOWERING LOCUS T
LCL: LHY/CCA1-Like
UBQ: POLYUBIQUITIN
ACT: ACTIN
WGD: whole-genome duplication
TRD: transposed duplication
TD: tandem duplication
PD: proximal duplication
DSD: dispersed duplication
MYA: million years ago
qRT–PCR: quantitative real-time PCR
ZT: zeitgeber time
GFP: green fluorescent protein
OE: overexpression

## References

[1] Inoue K, Araki T, Endo M (2018). Circadian clock during plant development. J Plant Res.

[2] Nohales MA, Kay SA (2016). Molecular mechanisms at the core of the plant circadian oscillator. Nat Struct Mol Biol.

[3] Harmer SL (2009). The circadian system in higher plants. Annu Rev Plant Biol.

[4] Gil KE, Park CM (2019). Thermal adaptation and plasticity of the plant circadian clock. New Phytol.

[5] Oakenfull RJ, Davis SJ (2017). Shining a light on the Arabidopsis circadian clock. Plant Cell Environ.

[6] Greenham K, McClung CR (2015). Integrating circadian dynamics with physiological processes in plants. Nat Rev Genet.

[7] Lu SX, Knowles SM, Andronis C, Ong MS, Tobin EM (2009). CIRCADIAN CLOCK ASSOCIATED1 and LATE ELONGATED HYPOCOTYL function synergistically in the circadian clock of Arabidopsis. Plant Physiol.

[8] Wang ZY, Tobin EM (1998). Constitutive expression of the CIRCADIAN CLOCK ASSOCIATED 1 (CCA1) gene disrupts circadian rhythms and suppresses its own expression. Cell..

[9] Schaffer R, Ramsay N, Samach A, Corden S, Putterill J, Carre IA, Coupland G (1998). The late elongated hypocotyl mutation of Arabidopsis disrupts circadian rhythms and the photoperiodic control of flowering. Cell..

[10] Mizoguchi T, Wheatley K, Hanzawa Y, Wright L, Mizoguchi M, Song HR, Carre IA, Coupland G (2002). LHY and CCA1 are partially redundant genes required to maintain circadian rhythms in Arabidopsis. Dev Cell.

[11] Nagel DH, Kay SA (2012). Complexity in the wiring and regulation of plant circadian networks. Curr Biol.

[12] Jung C, Muller AE (2009). Flowering time control and applications in plant breeding. Trends Plant Sci.

[13] Shim JS, Kubota A, Imaizumi T (2017). Circadian clock and photoperiodic flowering in Arabidopsis: CONSTANS is a hub for signal integration. Plant Physiol.

[14] Murakami M, Tago Y, Yamashino T, Mizuno T (2007). Comparative overviews of clock-associated genes of Arabidopsis thaliana and Oryza sativa. Plant Cell Physiol..

[15] Wang K, Bu T, Cheng Q, Dong L, Su T, Chen Z, Kong F, Gong Z, Liu B, Li M (2021). Two homologous LHY pairs negatively control soybean drought tolerance by repressing the abscisic acid responses. New Phytol.

[16] Tian L, Zhao X, Liu H, Ku L, Wang S, Han Z, Wu L, Shi Y, Song X, Chen Y (2019). Alternative splicing of ZmCCA1 mediates drought response in tropical maize. PLoS One.

[17] Kusakina J, Rutterford Z, Cotter S, Marti MC, Laurie DA, Greenland AJ, Hall A, Webb AA (2015). Barley Hv CIRCADIAN CLOCK ASSOCIATED 1 and Hv PHOTOPERIOD H1 are circadian regulators that can affect circadian rhythms in Arabidopsis. PLoS One.

[18] Dubos C, Stracke R, Grotewold E, Weisshaar B, Martin C, Lepiniec L (2010). MYB transcription factors in Arabidopsis. Trends Plant Sci.

[19] Rawat R, Takahashi N, Hsu PY, Jones MA, Schwartz J, Salemi MR, Phinney BS, Harmer SL (2011). REVEILLE8 and PSEUDO-REPONSE REGULATOR5 form a negative feedback loop within the Arabidopsis circadian clock. PLoS Genet.

[20] Carre IA, Kim JY (2002). MYB transcription factors in the Arabidopsis circadian clock. J Exp Bot.

[21] Du H, Wang YB, Xie Y, Liang Z, Jiang SJ, Zhang SS, Huang YB, Tang YX (2013). Genome-wide identification and evolutionary and expression analyses of MYB-related genes in land plants. DNA Res.

[22] Farinas B, Mas P (2011). Functional implication of the MYB transcription factor RVE8/LCL5 in the circadian control of histone acetylation. Plant J.

[23] Hsu PY, Devisetty UK, Harmer SL (2013). Accurate timekeeping is controlled by a cycling activator in Arabidopsis. Elife..

[24] Rawat R, Schwartz J, Jones MA, Sairanen I, Cheng Y, Andersson CR, Zhao Y, Ljung K, Harmer SL (2009). REVEILLE1, a Myb-like transcription factor, integrates the circadian clock and auxin pathways. Proc Natl Acad Sci U S A.

[25] Perez-Garcia P, Ma Y, Yanovsky MJ, Mas P (2015). Time-dependent sequestration of RVE8 by LNK proteins shapes the diurnal oscillation of anthocyanin biosynthesis. Proc Natl Acad Sci U S A.

[26] Li B, Gao Z, Liu X, Sun D, Tang W (2019). Transcriptional profiling reveals a time-of-day-specific role of REVEILLE 4/8 in regulating the first wave of heat shock-induced gene expression in Arabidopsis. Plant Cell.

[27] Kidokoro S, Hayashi K, Haraguchi H, Ishikawa T, Soma F, Konoura I, Toda S, Mizoi J, Suzuki T, Shinozaki K (2021). Posttranslational regulation of multiple clock-related transcription factors triggers cold-inducible gene expression in Arabidopsis. Proc Natl Acad Sci U S A.

[28] Bian S, Li R, Xia S, Liu Y, Jin D, Xie X, Dhaubhadel S, Zhai L, Wang J, Li X (2018). Soybean CCA1-like MYB transcription factor GmMYB133 modulates isoflavonoid biosynthesis. Biochem Biophys Res Commun.

[29] Li X, Wu T, Liu H, Zhai R, Wen Y, Shi Q, Yang C, Wang Z, Ma F, Xu L (2020). REVEILLE transcription factors contribute to the nighttime accumulation of anthocyanins in 'Red Zaosu' (Pyrus bretschneideri Rehd.) pear fruit skin. Int J Mol Sci.

[30] Chen S, Huang HA, Chen JH, Fu CC, Zhan PL, Ke SW, Zhang XQ, Zhong TX, Xie XM (2020). SgRVE6, a LHY-CCA1-like transcription factor from fine-stem stylo, upregulates NB-LRR gene expression and enhances cold tolerance in tobacco. Front Plant Sci.

[31] Wu J, Wang Z, Shi Z, Zhang S, Ming R, Zhu S, Khan MA, Tao S, Korban SS, Wang H (2013). The genome of the pear (Pyrus bretschneideri Rehd.). Genome Res.

[32] Fornara F, de Montaigu A, Coupland G (2010). SnapShot: control of flowering in Arabidopsis. Cell..

[33] Petersen J, Rredhi A, Szyttenholm J, Mittag M (2022). Evolution of circadian clocks along the green lineage. Plant Physiol.

[34] Cao ZH, Zhang SZ, Wang RK, Zhang RF, Hao YJ (2013). Genome wide analysis of the apple MYB transcription factor family allows the identification of MdoMYB121 gene confering abiotic stress tolerance in plants. PLoS One.

[35] Li X, Xue C, Li J, Qiao X, Li L, Yu L, Huang Y, Wu J (2016). Genome-wide identification, evolution and functional divergence of MYB transcription factors in Chinese white pear (Pyrus bretschneideri). Plant Cell Physiol.

[36] Cao Y, Han Y, Li D, Lin Y, Cai Y (2016). MYB transcription factors in Chinese pear (Pyrus bretschneideri Rehd.): genome-wide identification, classification, and expression profiling during fruit development. Front. Plant Sci.

[37] Zhang C, Ma R, Xu J, Yan J, Guo L, Song J, Feng R, Yu M (2018). Genome-wide identification and classification of MYB superfamily genes in peach. PLoS One.

[38] Liu J, Wang J, Wang M, Zhao J, Zheng Y, Zhang T, Xue L, Lei J (2021). Genome-wide analysis of the R2R3-MYB gene family in Fragaria x ananassa and its function identification during anthocyanins biosynthesis in pink-flowered strawberry. Front Plant Sci.

[39] Naing AH, Kim CK (2018). Roles of R2R3-MYB transcription factors in transcriptional regulation of anthocyanin biosynthesis in horticultural plants. Plant Mol Biol.

[40] Chaudhury A, Dalal AD, Sheoran NT (2019). Isolation, cloning and expression of CCA1 gene in transgenic progeny plants of japonica rice exhibiting altered morphological traits. PLoS One.

[41] Bian S, Jin D, Li R, Xie X, Gao G, Sun W, Li Y, Zhai L, Li X (2017). Genome-wide analysis of CCA1-like proteins in soybean and functional characterization of GmMYB138a. Int J Mol Sci.

[42] Zhang Z, Chen J, Su Y, Liu H, Chen Y, Luo P, Du X, Wang D, Zhang H (2015). TaLHY, a 1R-MYB transcription factor, plays an important role in disease resistance against stripe rust fungus and ear heading in wheat. PLoS One.

[43] Potter D, Eriksson T, Evans RC, Oh S, Smedmark JEE, Morgan DR, Kerr M, Robertson KR, Arsenault M, Dickinson TA, Campbell CS (2007). Phylogeny and classification of Rosaceae. Plant Syst Evol.

[44] Wang Y, Ficklin SP, Wang X, Feltus FA, Paterson AH (2016). Large-scale gene relocations following an ancient genome triplication associated with the diversification of core eudicots. PLoS One.

[45] Qiao X, Li Q, Yin H, Qi K, Li L, Wang R, Zhang S, Paterson AH (2019). Gene duplication and evolution in recurring polyploidization-diploidization cycles in plants. Genome Biol.

[46] Takata N, Saito S, Saito CT, Nanjo T, Shinohara K, Uemura M (2009). Molecular phylogeny and expression of poplar circadian clock genes, LHY1 and LHY2. New Phytol.

[47] Li C, Liu XJ, Yan Y, Alam MS, Liu Z, Yang ZK, Tao RF, Yue EK, Duan MH, Xu JH (2022). OsLHY is involved in regulating flowering through the Hd1- and Ehd1- mediated pathways in rice (Oryza sativa L.). Plant Sci.

[48] Liu C, Zhang Q, Dong J, Cai C, Zhu H, Li S (2022). Genome-wide identification and characterization of mungbean CIRCADIAN CLOCK ASSOCIATED 1 like genes reveals an important role of VrCCA1L26 in flowering time regulation. BMC Genomics.

[49] Song YH, Shim JS, Kinmonth-Schultz HA, Imaizumi T (2015). Photoperiodic flowering: time measurement mechanisms in leaves. Annu Rev Plant Biol.

[50] Song Y, Zhang R, Gao S, Pan Z, Guo Z, Yu S, Wang Y, Jin Q, Chen X, Zhang L (2022). Transcriptome analysis and phenotyping of walnut seedling roots under nitrogen stresses. Sci Rep.

[51] Wang L, Zhou A, Li J, Yang M, Bu F, Ge L, Chen L, Huang W (2021). Circadian rhythms driving a fast-paced root clock implicate species-specific regulation in Medicago truncatula. J Integr Plant Biol.

[52] Qing J, Dawei W, Jun Z, Yulan X, Bingqi S, Fan Z (2019). Genome-wide characterization and expression analyses of the MYB superfamily genes during developmental stages in Chinese jujube. PeerJ..

[53] Daccord N, Celton JM, Linsmith G, Becker C, Choisne N, Schijlen E, van de Geest H, Bianco L, Micheletti D, Velasco R (2017). High-quality de novo assembly of the apple genome and methylome dynamics of early fruit development. Nat Genet.

[54] Verde I, Abbott AG, Scalabrin S, Jung S, Shu S, Marroni F, Zhebentyayeva T, Dettori MT, Grimwood J, Cattonaro F (2013). The high-quality draft genome of peach (Prunus persica) identifies unique patterns of genetic diversity, domestication and genome evolution. Nat Genet.

[55] VanBuren R, Wai CM, Colle M, Wang J, Sullivan S, Bushakra JM, Liachko I, Vining KJ, Dossett M, Finn CE (2018). A near complete, chromosome-scale assembly of the black raspberry (Rubus occidentalis) genome. Gigascience..

[56] Edger PP, VanBuren R, Colle M, Poorten TJ, Wai CM, Niederhuth CE, Alger EI, Ou S, Acharya CB, Wang J (2018). Single-molecule sequencing and optical mapping yields an improved genome of woodland strawberry (Fragaria vesca) with chromosome-scale contiguity. Gigascience..

[57] Shirasawa K, Isuzugawa K, Ikenaga M, Saito Y, Yamamoto T, Hirakawa H, Isobe S (2017). The genome sequence of sweet cherry (Prunus avium) for use in genomics-assisted breeding. DNA Res.

[58] Zhang Q, Chen W, Sun L, Zhao F, Huang B, Yang W, Tao Y, Wang J, Yuan Z, Fan G (2012). The genome of Prunus mume. Nat Commun.

[59] Swarbreck D, Wilks C, Lamesch P, Berardini TZ, Garcia-Hernandez M, Foerster H, Li D, Meyer T, Muller R, Ploetz L (2008). The Arabidopsis information resource (TAIR): gene structure and function annotation. Nucleic Acids Res.

[60] Thompson JD, Gibson TJ, Plewniak F, Jeanmougin F, Higgins DG (1997). The CLUSTAL_X windows interface: flexible strategies for multiple sequence alignment aided by quality analysis tools. Nucleic Acids Res.

[61] Lee TH, Tang H, Wang X, Paterson AH (2013). PGDD: a database of gene and genome duplication in plants. Nucleic Acids Res.

[62] Chen C, Chen H, Zhang Y, Thomas HR, Frank MH, He Y, Xia R (2020). TBtools: an integrative toolkit developed for interactive analyses of big biological data. Mol Plant.

[63] Wang D, Zhang Y, Zhang Z, Zhu J, Yu J (2010). KaKs_Calculator 2.0: a toolkit incorporating gamma-series methods and sliding window strategies. Genomics Proteomics Bioinformatics.

[64] Bailey TL, Williams N, Misleh C, Li WW (2006). MEME: discovering and analyzing DNA and protein sequence motifs. Nucleic Acids Res.

[65] Hu B, Jin J, Guo AY, Zhang H, Luo J, Gao G (2015). GSDS 2.0: an upgraded gene feature visualization server. Bioinformatics..

[66] Lescot M, Dehais P, Thijs G, Marchal K, Moreau Y, Van de Peer Y, Rouze P, Rombauts S (2002). PlantCARE, a database of plant cis-acting regulatory elements and a portal to tools for in silico analysis of promoter sequences. Nucleic Acids Res.

[67] Bustin SA, Benes V, Garson JA, Hellemans J, Huggett J, Kubista M, Mueller R, Nolan T, Pfaffl MW, Shipley GL (2009). The MIQE guidelines: minimum information for publication of quantitative real-time PCR experiments. Clin Chem.

[68] Xie Q, Wang P, Liu X, Yuan L, Wang L, Zhang C, Li Y, Xing H, Zhi L, Yue Z (2014). LNK1 and LNK2 are transcriptional coactivators in the Arabidopsis circadian oscillator. Plant Cell.

[69] Sparkes IA, Runions J, Kearns A, Hawes C (2006). Rapid, transient expression of fluorescent fusion proteins in tobacco plants and generation of stably transformed plants. Nat Protoc.

[70] Clough SJ, Bent AF (1998). Floral dip: a simplified method for Agrobacterium-mediated transformation of Arabidopsis thaliana. Plant J.

