# Supplementary material for: Characterization of the REVEILLE family in Rosaceae and role of PbLHY in flowering time regulation
Source: BMC Genomics. 2023 Jan 28;24:49. doi: 10.1186/s12864-023-09144-4 (PMC9883883; doi:10.1186/s12864-023-09144-4)
Supplement: Supplementary file 1 — Additional file 1: Fig. S1. (a) Coding sequence of PbLHY. (b) Alignment of PbLHY and AtCCA1/AtLHY protein sequences. The red box indicates the MYB domain. The blue backgrounds correspond to the percent identity of the multiple alignment. Fig. S2. Alignment of conserved domains from PbRVE proteins. (a) Alignment of MYB domains from ten PbRVEs and eleven AtRVEs. Red box indicates MYB domain, and blue box indicates SHAQK(Y/F) F sequence. (b) Alignment of LCL domains from subfamily II members. The red box indicates the MYB domain. The green box indicates the LCL domain. The blue backgrounds correspond to the percent identity of the multiple alignment. Fig. S3. The motif details of ten conserved domains identified in RVE proteins and the logos of these domains created using the MEME. Amino acids are expressed in the standard single letter code. The size of the letters at each position represents their frequency. [file 12864_2023_9144_MOESM1_ESM.pdf]

a

>PbLHY coding sequence

ATGGACACAAACTCATCTGGAGAAGACCTTTTCATCAAGGCACGAAAGCCTTATACGATTACCAAGCAACGAGAGCGATGGACAGAGGAGGAGCATAATAGGTTTCT
TGATGCCTTGAAGCTCTATGGCCGAGCATGGCAGCGCATTGAAGAACATATTGGAACGAAGACTGCTGTGCAGATCAGGAGTCATGCTCAGAAGTTCTTTTCAAAGC
TGGAGAAGGAGGCACACAATAAAGGTGTTCCAATGGGACAATCAATTGACATAGATATCCCGCCTCCACGTCCCAAGAGGAAACCAAGCAATCCTTACCCTCGAAAG
TCTAGTTCAGCTGCCACGACATTGACCAATCCGCATGTGGGAGCAAAGGATGGAAAACTTTTATCTACAGCATCTTCTTCACACTGTCCACAAGTAGTGGATTTGGAG
AAAGAACCACCTCATGAGAGACCTACTGAAGAAGAAAATCCGAAAAATGGAAAAGAAAATCCGGATGAAAATTGCTCAGAAGCCTTCACTTTGCTCCAAGAACATAAT
TGTTCCTCTGATTCTACTGCAAACAAAAATTCTATACCTACACAGGTGGTGCTGAGAAACGCTTGCACTTTTCAGGAGTTTGTGCCTTCGCTGAAAGAGGTAATAAGT
CAAGATGTAACAACTGAATCTTATGTTACTATTGAACTTAATGGAAATCAGAATATGAAGAAAAATGATGCCAAAAAGATAGTTCAAGTTAATGGCACAAGTGGAGCCT
CAGTGTCAGAGAACACTAATGCTCTTCATAAGAAGTTGGTTCAAGGTGAGAAAGCAGATGATTTGAATTGTGCATTGCCAACAGATGGGATGCAAAGCACTCAGAAC
TACCCAAGGAATGTTCCTGTACATGTACTGGATGGTAGCGTAGGAGCGTGTAATCAAACTACACCTGCAGATATGTCATTCCCTGACTCCGGTTTCCATCCTATGGG
GAAGGTTCATGGACAGCCTAACCTGTTTGCAAATCCAGCGGCATCTACTACTACTGAACATGAAAGTAATGCATCAAGATCTACTATTCACCAATCGTTTCCAGCTTTT
CACCCTCCCTTCACACCATTCCACCATTGTCAAAAGGACTACCAATCATTTCACCACATTTCCTCAACATTTTCTAGCCTTATTGTGTCGTCTCTGCTACAAAACCCTG
CAGCCCATGCTGCGGCTAGCTTTGCAGCCACATTTTGGCCTTATGCAAATGTGGAAAATACGGAAGACTCTCCTGCATGCTCCTCTGCTGGAGGTTTTCCACCGAAC
TCCCCTCCAAGTATGGCAGCAATTGCTGCTGCCACTGTAGCTGCTGCAACTGCATGGTGGGCAGCCCATGGATTGCTTCCCTTGTGCGCTCCTGTTCAAACTTCTTT
GAGCTGCCCTCCCATGCCCATGACTGGGGGGGTTCCGTCAGTGAATACTGATCAAGATCCTGCAGTGATGACAGAGAGAGGCGAGAATTCTCTTCAAATTCCTTCT
GCAGAGGATCAACAATTGGAGCCGGAACACTTGGAAGCTGTGGATGCTCAGCATTTAGCTTCACAATCACCAAACAGGTCATCATCACACTCTGATAATGGAGGTGC
AGAGCCTAATACAGAATTGAAGTCTGCTGATGATGAGAAGGCTGTAGCATCAACTGCTCGAGTTAATGATTCAGACAATGCAAAGAGTAGAAAACAGGTTGACCATT
CTTCGTGTGGCTCCAACACACCTTCCGGCAGTGAAGTAGAGACAGATGCATTAGAGAAGCATGAGAAAGGCACAGAAGAACCAGAAGAACCTAAAGAACCAAAAGA
ACTTGATCTAAATCTCTTAGCATCTGATTCTAATTATCGTCGCAGTAGAAGTATCAGCAACGTGAATGATCCATGGAAAGAGGTTTCTGAAGAGGGCCGGATGGCCTT
TCAAGCGCTATTCGCAAGAGAGGTACTGCCCCAGAGTTTTTCACCGCCTTCACACTGCCTCAAGGTTAATGAGCATCAGGATGCTACAGAAGCAGCAAATCAAAATT
CTGACAAGAAAGATAGAGATGCATCTGCATCACAATTTGACTTCTATTCGTGGATGTCATTTCCGTATCCTCCAGAAGCGGAGAAAAGTGTGTCACCAGCACAAGGC
AGCAACACAGAAGGGCTACTGACACTAGGACTTAGCCAAGGGAAACTCAAGGCCCGCCGAACAGGATTCAAACCTTACAAGAGGTGCTCCGTAGAGGCCAACGAG
AACCGGGTGGCCAATGCCAGCAGCCACTGCGAAGAGAAGTGCCCGAAGAGGTTACGCTTAGAAGGGGAAGCTCAAACT

b

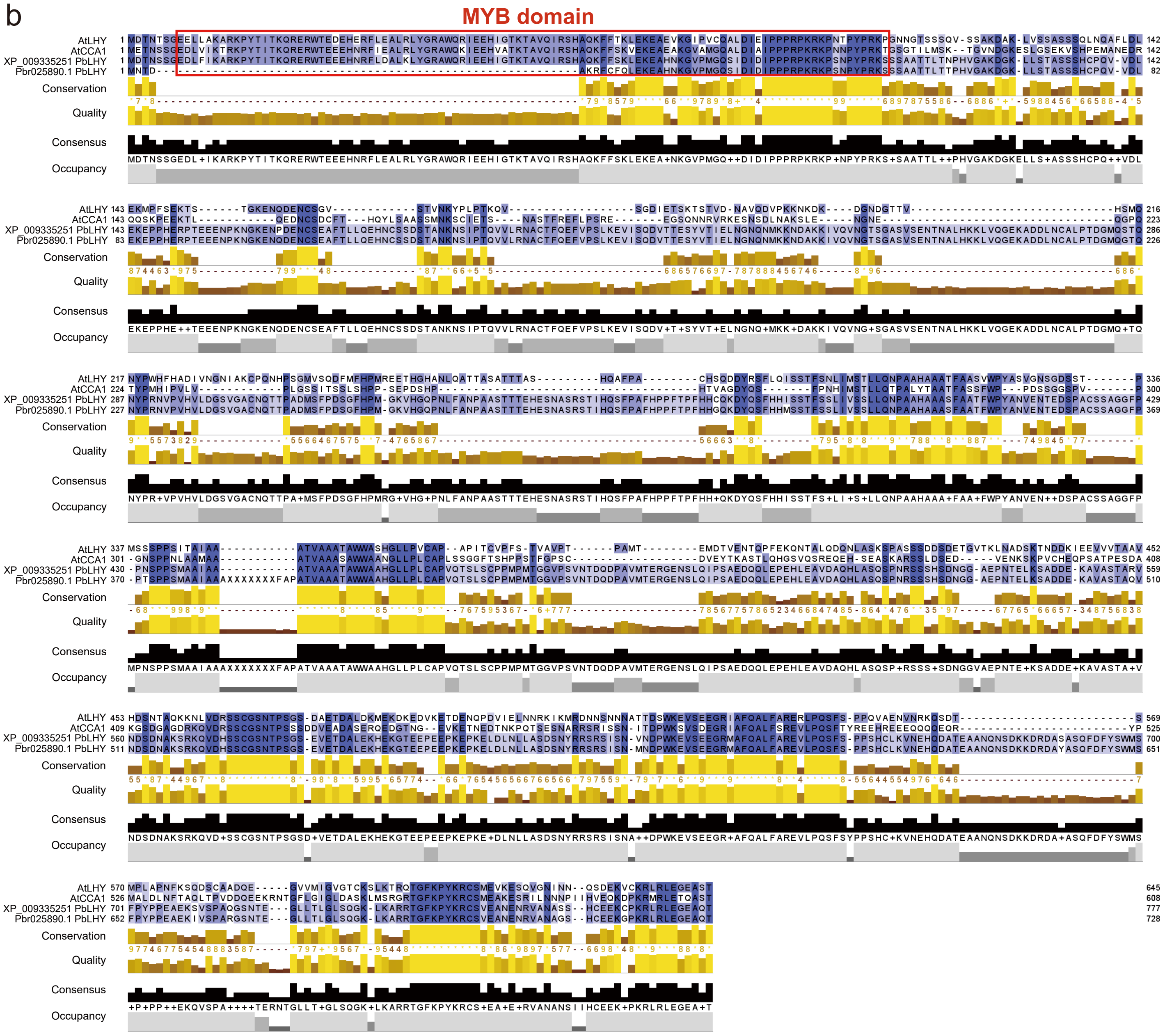

Fig. S1 (a) Coding sequence of *PbLHY*. (b) Alignment of PbLHY and AtCCA1/AtLHY protein sequences. The red box indicates the MYB domain. The blue backgrounds correspond to the percent identity of the multiple alignment.

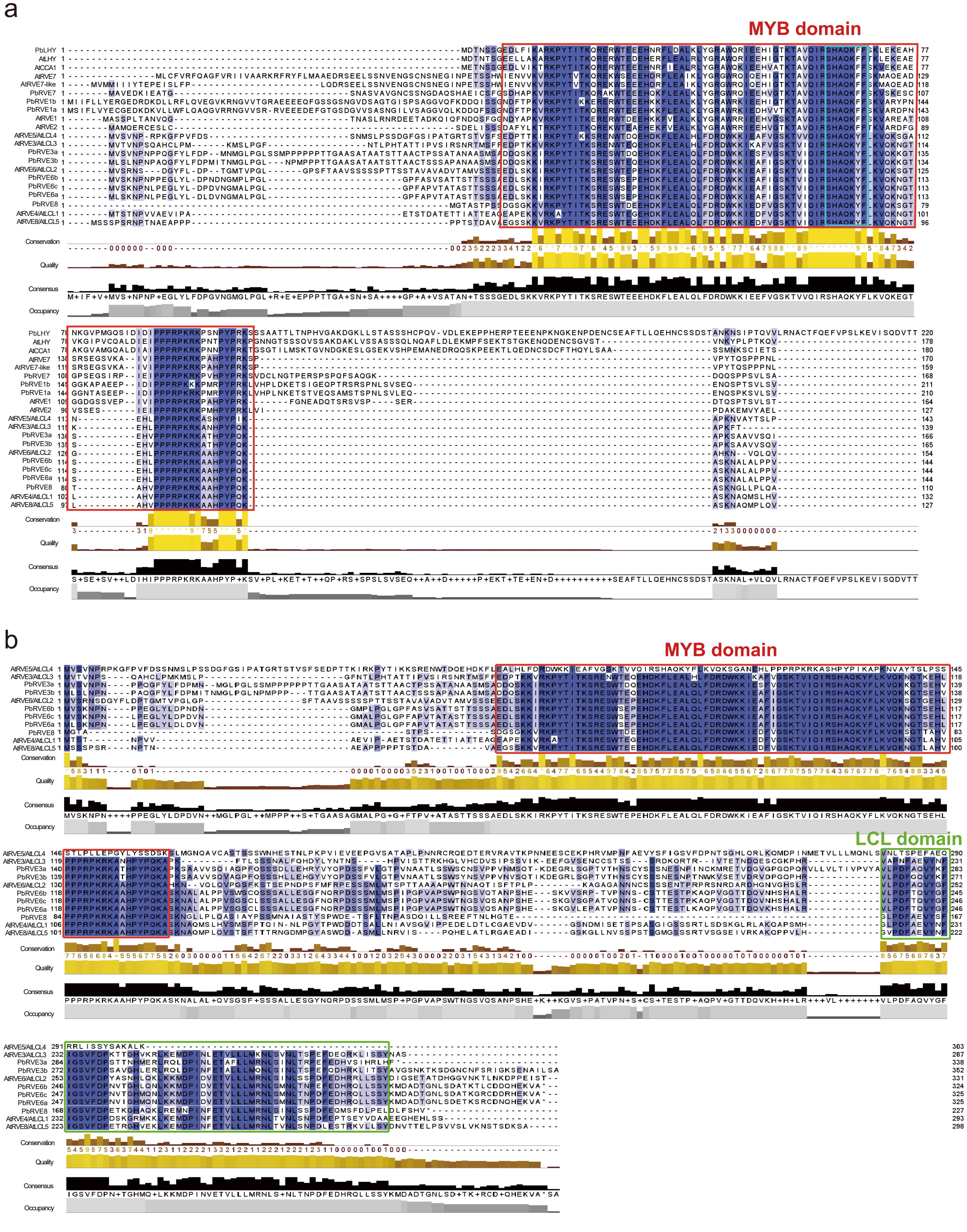

Fig. S2 Alignment of conserved domains from PbRVE proteins. (a) Alignment of MYB domains from ten PbRVEs and eleven AtRVEs. Red box indicates MYB domain, and blue box indicates SHAQK(Y/F)F sequence. (b) Alignment of LCL domains from subfamily II members. The red box indicates the MYB domain. The green box indicates the LCL domain. The blue backgrounds correspond to the percent identity of the multiple alignment.

		E-value	Sites	Width
Motif 1	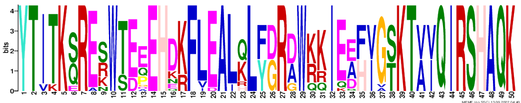	3.8e-835	21	50
Motif 2	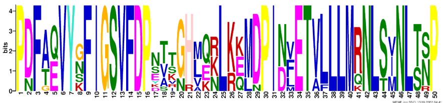	3.9e-329	11	50
Motif 3	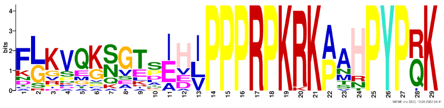	3.7e-311	21	29
Motif 4	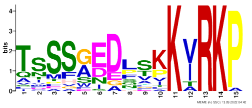	2.8E-74	21	15
Motif 5	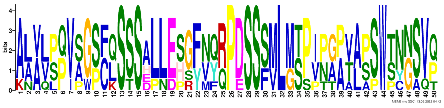	3E-64	6	50
Motif 6	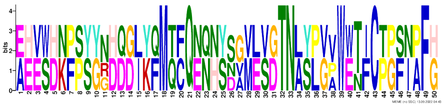	4.2E-44	4	50
Motif 7	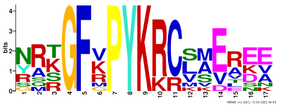	6.5E-44	10	17
Motif 8	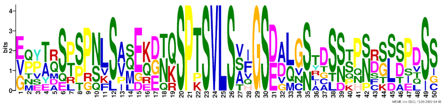	2.6E-29	7	50
Motif 9	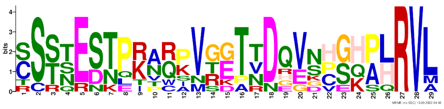	1.6E-30	8	29
Motif 10	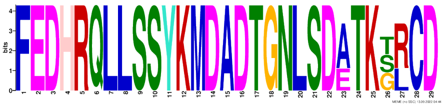	5E-25	3	29

Fig. S3 The motif details of ten conserved domains identified in RVE proteins and the logos of these domains created using the MEME. Amino acids are expressed in the standard single letter code. The size of the letters at each position represents their frequency.
